# Usefulness of a new anthropomorphic phantom simulating the chest and abdomen regions in PET tests

**DOI:** 10.1007/s12149-024-02007-2

**Published:** 2024-12-07

**Authors:** Hiroaki Sagara, Kazumasa Inoue, Chikara Mano, Hironori Kajiwara, Yuichi Nagai, Hirofumi Fujii, Anri Inaki

**Affiliations:** 1 Department of Radiological Technology, Faculty of Health Sciences, Tsukuba International University, 6-20-1, Manabe, Tsuchiura, 300-0051 Japan; 2 Department of Radiological Sciences, Graduate School of Human Health Sciences, Tokyo Metropolitan University, 7-2-10 Arakawa‑ku, Tokyo, 116‑8551 Japan; 3 Division of Functional Imaging, Exploratory Oncology Research and Clinical Trial Center, National Cancer Center, 6-5-1 Kashiwanoha, Kashiwa, 277-8577 Japan; 4 Department of Radiologic Technology, National Cancer Center Hospital East, 6-5-1 Kashiwanoha, Kashiwa, 277-8577 Japan

**Keywords:** PET, Anthropomorphic phantom, Count rate, Image quality

## Abstract

**Objective:**

To investigate the clinical utility of a new anthropomorphic phantom that reproduces the chest and abdomen better than the conventional National Electrical Manufacturers Association (NEMA) body phantom, count rates and image quality of PET images obtained from patients were evaluated.

**Methods:**

Anthropomorphic phantoms were used to include radioactivity in the lung, liver, kidney, and background regions. Two NEMA body phantoms were used for chest and abdominal assessments. The cross calibration factor (CCF) cylinder phantom was also used to reproduce the distribution of radioactivity outside the field of view, simulating the patient brain. Four types of phantoms were used in the PET imaging experiment, and for each phantom, the prompt coincidence count rates, random coincidence count rates, true + scatter coincidence count rates, and single photon count rates were measured. Then, these count rates were compared with count rates from actual clinical data. PET image quality assessment was done using the parameters, noise equivalent count patient (NEC_patient_), noise equivalent count density (NEC_density_), and liver signal-to-noise ratio (SNR).

**Results:**

Random coincidence count rates showed that the data obtained from each phantom were in good agreement with the clinical data. True + scatter coincidence count rates had better agreement with clinical data when measured for anthropomorphic phantoms than for the NEMA body phantoms. Furthermore, when the CCF Cylinder phantom simulating the brain was placed outside the imaging field of view, the results were closer to the clinical data. PET image quality was 1.4% higher for NEC_patient_ obtained from anthropomorphic phantoms compared to the mean obtained from clinical data. NEC_density_ was 15.0% lower than the mean value obtained from clinical data. Liver SNR was 14.8% higher in PET images reconstructed using the 3D-ordered subsets expectation maximization (OSEM) method. It was 10.0% lower in PET images reconstructed with the image reconstruction method Q.Clear (GE Healthcare) using the Bayesian penalized likelihood (BPL) method.

**Conclusion:**

The new anthropomorphic phantom was more consistent with the count rates obtained from clinical data than the conventional NEMA body phantoms were and it was able to better simulate the distribution of radioactivity concentrations in the patients by reproducing the distribution of radioactivity concentrations outside the field of view.

## Introduction

Positron emission tomography/computed tomography (PET/CT) using ^18^F-fluorodeoxyglucose (FDG) plays a major role in diagnosing malignant lesions and determining treatment efficacy [1–3]. Recently, with the advent of molecular targeted therapies and immunotherapy, tumor metabolic activation is expected to be quantitatively assessed by FDG-PET imaging [4]. FDG-PET is therefore going beyond an imaging tool to become a quantitative imaging biomarker [5]. However, PET image quality and quantifiability of the standardized uptake value (SUV) are significantly influenced by many factors, including injection dose, uptake time, subject physique, PET scanner performance, and image reconstruction parameters [6]. In particular, the detectability of small lesions and the SUV of tumors easily varies depending on these factors. These variations may affect the diagnostic results of malignant lesions obtained with a single PET scanner and, in multicenter studies using multiple PET scanners, they may significantly reduce the reliability of the study results obtained [7]. Therefore, multicenter studies of tumor FDG-PET need to validate and standardize imaging protocols, image quality, and quantitation with an appropriate phantom before any study is begun.

Several organizations, including the Quantitative Imaging Biomarkers Alliance (QIBA) and the Society of Nuclear Medicine and Molecular Imaging (SNMMI), have recommended the National Electrical Manufacturers Association (NEMA) NU-2 image quality phantom as a standard for PET image quality and the quantification of SUV [8, 9]. Furthermore, the NEMA body phantom is frequently used to optimize imaging protocols and validate image reconstruction techniques, as well as to evaluate the performance of newly developed PET scanners [10]. However, its simple structure makes reproduction difficult of a non-uniform radioactivity concentration distribution similar to that of the patient’s distribution. Therefore, even if the radioactivity concentration enclosed in the phantom is the same as in the patient, the counting rate characteristics obtained are very different from those of actual clinical data [11, 12]. To solve these problems, Matheoud et al. [13] reproduced the count rates obtained from patients by placing thick scatterers around the NEMA body phantom. Focusing on the pelvic region of the patients, Inoue et al. [14] developed a pelvic anthropomorphic phantom that reproduced the bone structures which absorb gamma radiation emitted from the pelvic cavity and the bladder, the latter of which has extremely high physiological radioactivity. As a result, this improved NEMA body phantom was able to accurately reproduce the count rates obtained from clinical data and simulate actual clinical conditions in the pelvis. However, although these anthropomorphic phantoms were able to match the count rates obtained from patients, the PET image quality assessment obtained from patients and phantoms did not use the same image quality metrics. Doshi et al. [12] reported an 8.7% reduction in contrast ratio when image quality was assessed using anthropomorphic phantoms, compared to results obtained in uniform phantom experiments. This suggests that the experimental results obtained with the conventional NEMA body phantom may be overestimations.

SNMMI developed the anthropomorphic chest phantom as an easy-to-use PET image quality assessment tool for all facilities, from the fabrication of a new phantom to assessment of imaging and image quality; this phantom is compatible with the Clinical Trials Network (CTN) [15]. However, despite the similarity of the phantom in structure to the human chest, no validation has been reported to match the count rate to the patient; in other words, the phantom count rate may not adequately reproduce actual clinical situations. In addition, as only the thoracic region of the human chest is simulated, the phantom may not be suitable for assessing image quality in areas such as the liver, where the radioactivity concentration is relatively higher than in the lung field.

In this study, the clinical usefulness of a new anthropomorphic phantom, which reproduces clinical conditions in the chest and abdominal regions, was determined by matching the count rates obtained from patients and evaluating the PET image quality obtained using the same image quality metrics as those applied for patients.

## Materials and methods

### Phantoms

Four types of phantoms were used in this study: two NEMA body phantoms (denoted as NEMA), the thorax phantom (denoted as Thorax; manufactured by Kyoto Kagaku Co. Ltd., Kyoto, Japan), the NEMA_Cross calibration factor (CCF) Cylinder phantom (denoted as NEMA_Brain), and the Thorax_CCF Cylinder phantom (denoted as Thorax_Brain).

Thorax used was able to contain radioactivity in the lung, liver, kidney, and background areas. Alone, it weighed 21.0 kg, and when filled with solution, it weighed 37.5 kg. The CT value of each organ was 75 HU and the density was 1.13 g/cm^3^. The CT value of the bone equivalent material of Thorax was 370 HU, with a density of 1.29 g/cm^3^, while the area simulating the lung field was −900 HU, with a density of 0.30 g/cm^3^.

Thorax and two NEMA body phantoms were used to validate count rate characteristics and image quality assessment. Each NEMA body phantom consisted of six spheres with inner diameters of 10, 13, 17, 22, 28, and 37 mm and a cylindrical structure was inserted to simulate a lung according to the NEMA standards. The NEMA_Brain, used in quality control of PET scanners, was also used to reproduce the out-of-field radioactivity distribution to simulate the human brain.

### Patients

From among 96 patients who underwent ^18^F-FDG-PET/CT testing between 14 and 30 April 2020, data from 51 patients were analyzed in this study. The reasons for exclusion of 45 patients from the analysis were having an injection dose other than 3.7 MBq/kg ± 10%, an acquisition time other than 120 s per bed position (of which there were two positions), an uptake time < 50 min, blood glucose level > 150 mg/dL, and other types of missing data. Characteristics of the patients selected for analysis and the ^18^F-FDG-PET/CT testing conditions are listed in Table 1. The present study was approved by the Ethical Review Committee of the National Cancer Center (Study No. 2023–069). Due to its retrospective design, the requirement for informed patient consent was waived.

**Table 1:** Characteristics of 51 patients for the analysis

Parameters	Mean ± SD	Range
Age (yr)	67.8 ± 11.0	32–87
Body height (m)	1.66 ± 0.1	1.58–1.77
Body weight (kg)	62.7 ± 6.2	55–75
BMI (kg/m^2^)	22.8 ± 2.4	18.1–29.7
Injection dose (MBq)	222.2 ± 22.4	186.2–273.1
Dose/weight (MBq/kg)	3.54 ± 0.1	3.34–3.98
Blood glucose level (mg/dL)	99.4 ± 13.0	69–135
Uptake time (min)	64.6 ± 7.8	53–87

**Fig. 1:**
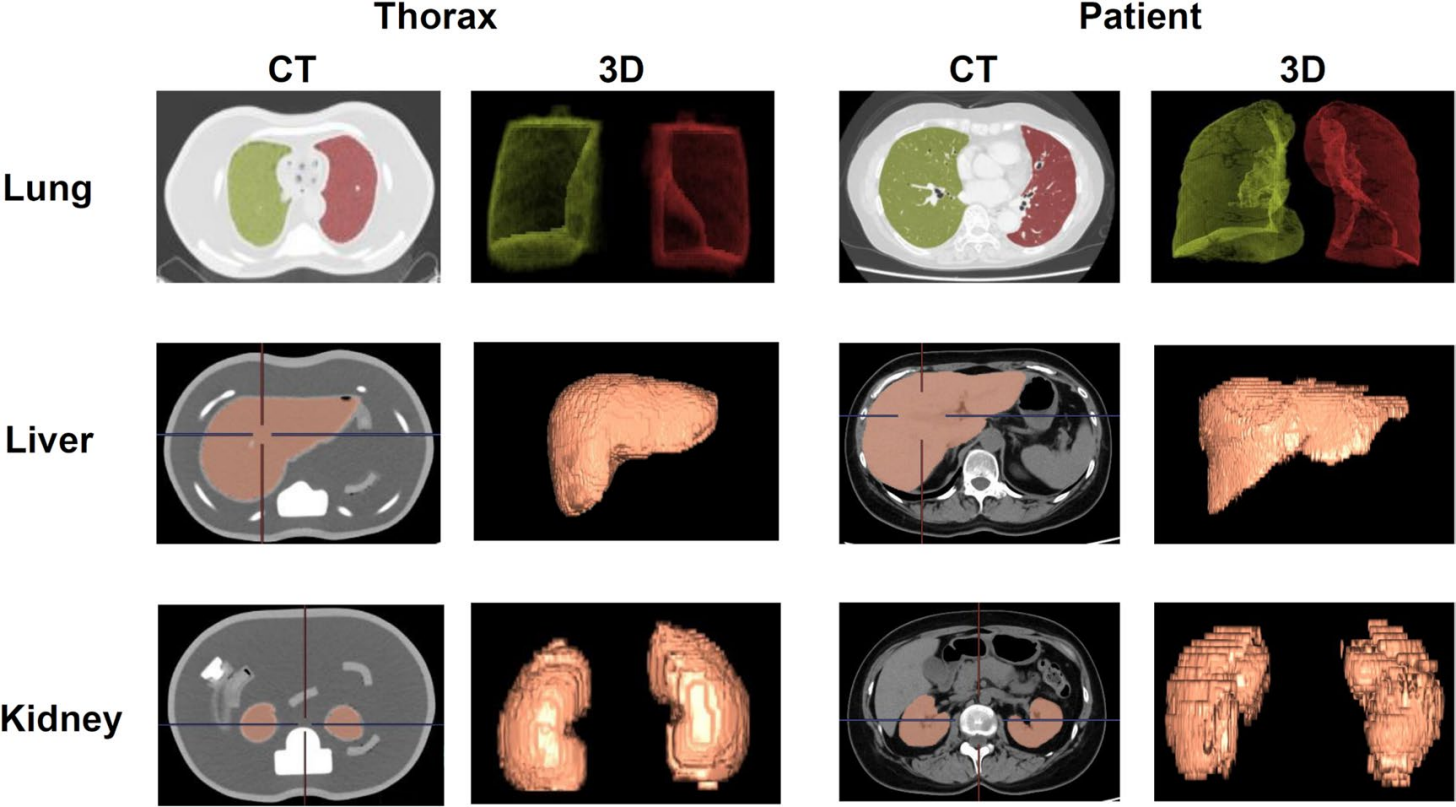
Example set of CT and 3D images of each organ obtained for one patient and the anthropomorphic Thorax phantom

To demonstrate the similarity of the Thorax phantom to real human body structures, the volumes of the lungs, liver, and kidneys were measured using CT images of 51 patients selected for the analysis. An example set is reproduced in Fig. 1. The software Synapse Vincent system Ver 5.4 (Fujifilm Corporation, Tokyo, Japan) was used to automatically measure each organ based on CT values. FDG-PET images were used to determine the radioactivity in the organs. The image information unification system ShadeQuest (Yokogawa, Tokyo, Japan) was used for this purpose.

For the PET/CT scanning, ^18^F-FDG was administered at 3.7 MBq/kg and imaging was started 60 min later. A CT scan was performed first. The CT scan was performed with a tube voltage of 120 kV and a tube current adjusted using auto exposure control (AEC) to achieve a standard deviation of 20 for CT values, with a rotation time of 0.6 s. The CT scan was followed by a whole-body PET scan in 3D mode. PET scan acquisition time was 120 s per bed position in the list mode of 3D acquisition.

### PET/CT scanner

The PET/CT scanner used was a Discovery IQ 5ring (GE Healthcare, Milwaukee, WI, USA). The detector of this scanner comprised Bi_4_Ge_3_O_12_ (BGO) crystals measuring 6.3 mm × 6.3 mm × 30 mm. The field of view (FOV) along the body axis was 260 mm and 79 images could be obtained in one scan. The energy window was 425–650 keV and the coincidence timing window was 9.5 ns. The number of overlaps between each bed position was 19, with an overlap percentage of 24%. The PET/CT instrument performance was measured according to NEMA NU-2 2012, with a system sensitivity of 20.1 cps/kBq at the center of the FOV and a scatter fraction of 37.9% [16].

### Phantom measurement experiment

**Fig. 2:**
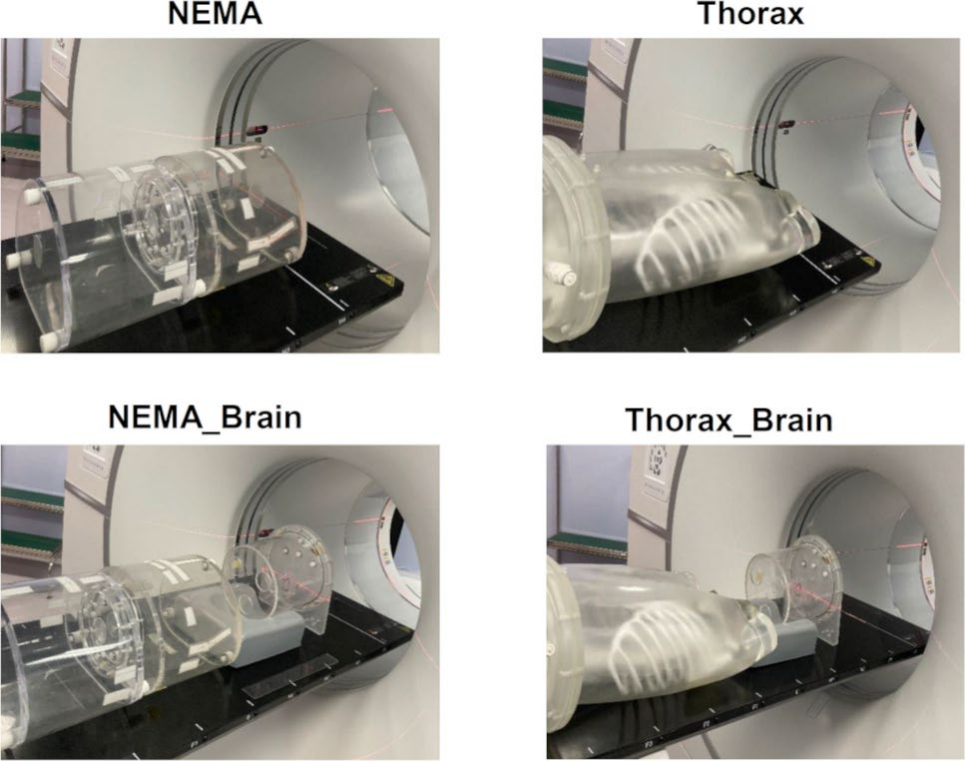
Geometric position in each phantom PET imaging

The four types of phantoms shown in Fig. 2 were used in the measurement experiment. The radioactivity of the injected dose enclosed in the NEMA body phantom was determined with reference to the guidelines for tumor FDG-PET imaging methods [17]. Two NEMA body phantoms were used to assess the chest and abdomen regions, with an injection dose of 110 MBq (5.39 kBq/mL). The ^18^F-FDG injection dose of the other phantoms was determined based on measured radioactivity in patients. The injection dose for Thorax phantom was 105.6 MBq (2.82 kBq/mL) and the injection dose for the CCF Cylinder phantom was 50 MBq (8.62 kBq/mL).

For all phantoms, the imaging conditions for CT data used for attenuation correction were set to a tube voltage of 120 kV and a tube current adjusted using AEC to achieve a standard deviation of 20 for CT values, with a rotation time of 0.6 s, as in actual clinical conditions. PET scans had acquisition times of 120 s per bed position in list mode with 3D acquisition as in clinical conditions. The imaging area of the PET and CT scan was the chest and abdomen area (bed position-2) of the human body, and the acquisition of this bed position-2 was repeated 20 times. The total acquisition time was 80 min.

### Data analysis for coincidence count rates

Prompt coincidence count rate (*R*_p_), random coincidence count rate (*R*_r_), and single photon count rate (*R*_sp_) were obtained from phantom data and raw patient data. True coincidence count rate + scatter coincidence count rate (*R*_t+s_) was calculated using Eq. (1)

1 $$R_{{{\text{t}} + {\text{s}}}} = R_{{\text{p}}} - R_{{\text{r}}} .$$

Here, *R*_p_, *R*_r_, and *R*_t+s_ were correlated with *R*_sp_ obtained from phantom and patient data. These count rates were approximated by a quadratic function. The mean *R*_sp_ in the phantom and patient data was calculated in three regions: chest, abdomen, and chest to abdomen. Random fraction (RF) was calculated from Eq. (2)

2 $${\text{Random}}\;{\text{fraction}}\left( \% \right) = \frac{{R_{{\text{r}}} }}{{R_{{\text{p}}} }} \times 100.$$

### Image quality

PET images were reconstructed from raw data obtained from phantom and patient data. Image reconstruction was performed using the 3D-ordered subsets expectation maximization (OSEM) method and the Bayesian penalized likelihood (BPL) method. OSEM reconstruction parameters were 12 subsets and 3 iterations with a 4 mm Gaussian filter for post-filtering. The BPL method set the penalty parameter, β-value, at 350. These parameter conditions are used in routine practice.

To compare the image quality of PET images of Thorax phantom and patients, image quality was assessed using NEC patient (NEC_patient_), NEC density (NEC_density_) and liver signal-to-noise ratio (liver SNR) [17]. The calculation of these image quality metrics was made using PETquactIE Ver. 3 (Nippon MediPhysics Co., Ltd.) [18].

NEC_patient_ was calculated using Eq. (3)

3 $${\text{NEC}}_{{{\text{patient}}}} = \frac{{\mathop \sum \nolimits_{i = 1}^{n} {\text{NEC}}_{i} }}{x/100}\left[ {{\text{Mcounts}}/{\text{m}}} \right].$$

Here, *NEC*_*i*_ and *x* represent the NEC for bed position-1 and bed position-2. The brain and bladder areas were excluded from the analysis scope due to the high physiological accumulation of FDG. The bed position-2 from chest to the abdomen was assessed.

NEC_i_ was calculated using Eq. (4)

4 $${\text{NEC}}_{i} = \left( {1 - {\text{SF}}} \right)^{2} \frac{{\left( {P_{i} - R_{i} } \right)^{2} }}{{\left( {P_{i} - R_{i} } \right) + \left( {1 + k} \right)R_{i} }}\left[ {{\text{Mcounts}}/{\text{m}}} \right].$$

Here, *P*_*i*_ and* D*_*i*_ represent the prompt coincidence count and random coincidence count, respectively, at the bed position *i* (*i* = 1 or 2). *SF* is the scatter fraction 0.37, based on the NEMA standard [16].* k* is a coefficient based on the correction method for contingent coincidences (1 for delayed coincidence measurements, 0 otherwise).

NEC_density_ was calculated using Eq. (5)

5 $${\text{NEC}}_{{{\text{density}}}} = \frac{{\mathop \sum \nolimits_{i = 1}^{n} {\text{NEC}}_{i} }}{V}\left[ {\frac{{{\text{kcounts}}}}{{{\text{cm}}^{3} }}} \right].$$

Here, *V* is the volume of the chest and abdomen region excluding the brain and bladder. The volume was calculated using PETquactIE Ver. 3. The calculation method was analyzed from CT data acquired according to the guidelines [17]. NEC_*i*_ was also calculated using data obtained from the chest and abdomen region.

Liver SNR was calculated using Eq. (6)

6 $${\text{Liver}}\;{\text{SNR}} = \frac{{C_{{{\text{liver}}}} }}{{{\text{SD}}_{{{\text{liver}}}} }}.$$

**Fig. 3:**
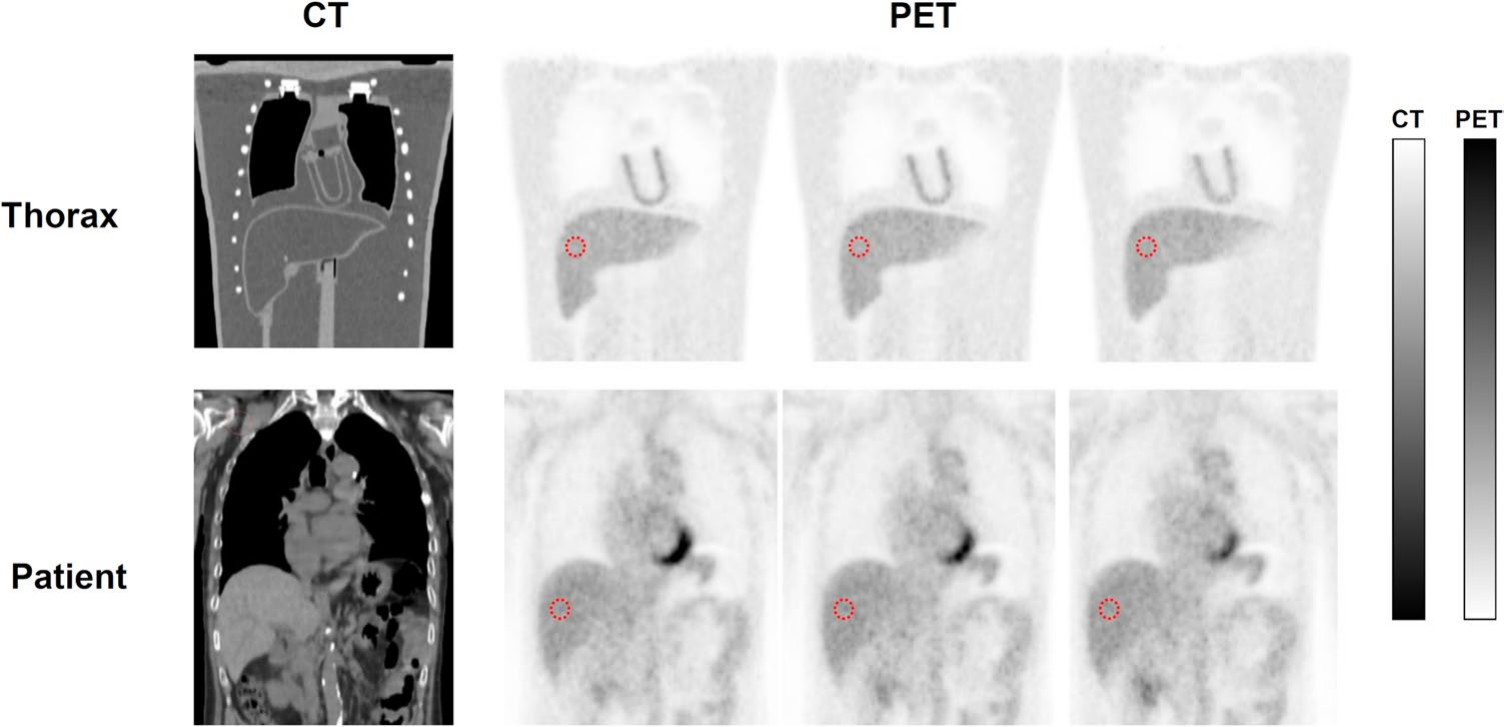
Placement of ROIs when calculating liver SNR obtained for patients and anthropomorphic thorax phantom

Here,* C*_liver_ is the mean of the counts in the region of interest (ROI) and SD_liver_ is the standard deviation of the counts in the ROI. The ROI was a circle of 3 cm diameter in the liver of the PET image, one in each of three consecutive slices (Fig. 3).

## Results

### Comparison of coincidence count rates of four types of phantoms and patients

The volumes of five regions in the analyzed patients were measured for comparison with the volumes of these organs included in Thorax phantom and the results are shown in Table 2. The size of each organ in the human body was larger than that in Thorax phantom, with the exception of the liver. The radioactivity of FDG enclosed in each organ of Thorax phantom was based on the radioactivity of each patient organ as follows: right lung 2.4 kBq/mL, left lung 2.4 kBq/mL, liver 12.0 kBq/mL, right kidney 32.0 kBq/mL, left kidney 32.0 kBq/mL, and background 4.0 kBq/mL. The radioactivity in the background region of the Thorax was designed for standard-sized adults patients and was set at 4.0 kBq/mL based on guidelines [17]. This concentration was also determined by assuming that the radiopharmaceuticals would be uniformly distributed throughout the whole body.

**Table 2:** Volumes of five regions for the Thorax phantom and patients (*n* = 51)

Region volume (mL)	Thorax phantom	Patients (*n* = 51) mean ± SD
Right lung	1167.4	1712.5 ± 407.9
Left lung	1053.4	1414.5 ± 411.6
Liver	1480.0	1270.0 ± 240.6
Right kidney	127.7	153.6 ± 38.5
Left kidney	122.8	164.3 ± 41.8

**Fig. 4:**
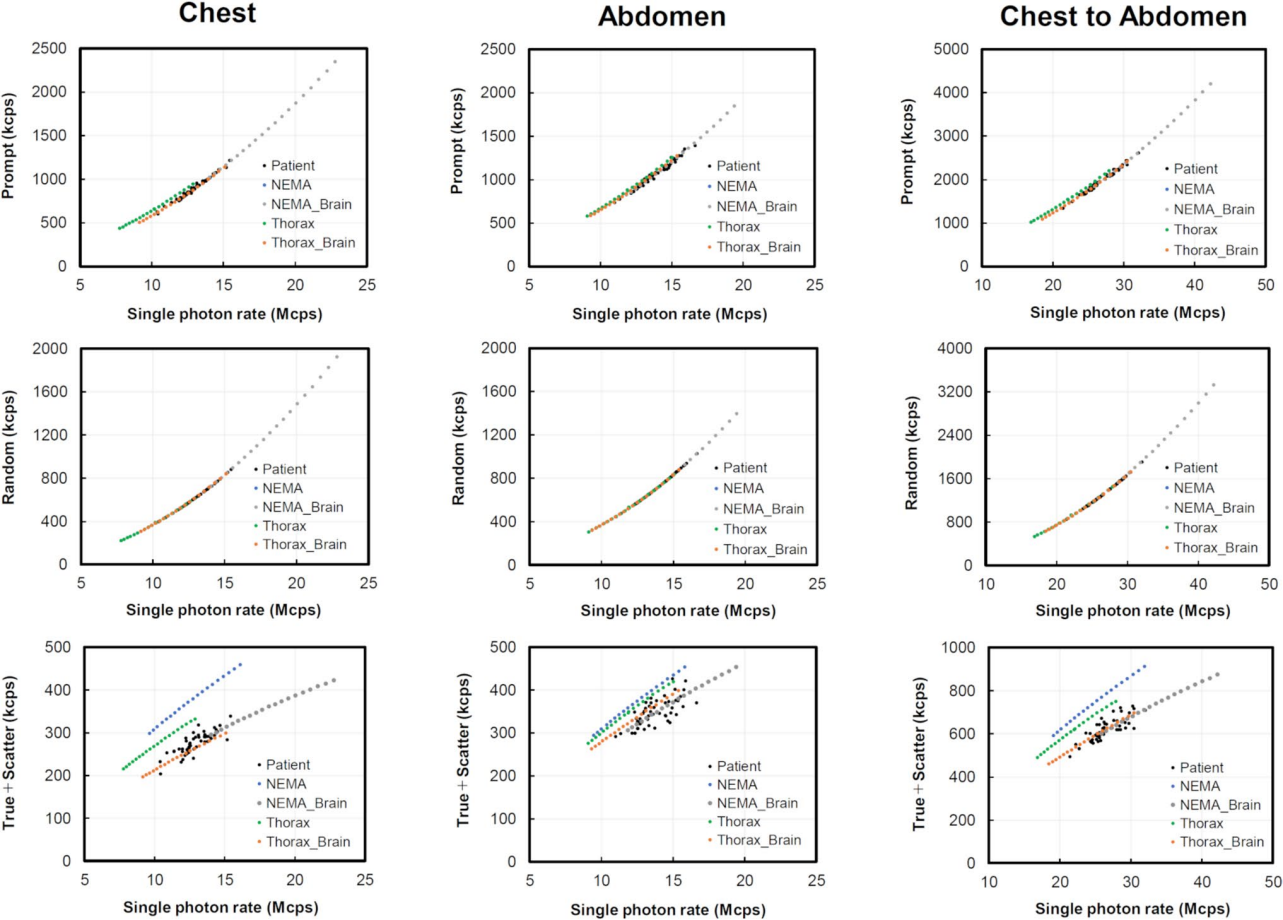
Correlation between the single photon rates of each phantom type and the prompt coincidence count rate, random coincidence count rate, and true + scatter coincidence count rate

The *R*_p_, *R*_r_, and *R*_t+s_ were correlated with *R*_sp_ obtained from phantom and patient data are shown in Fig. 4 for comparison. The *R*_p_ and *R*_r_ of each type of phantom agreed well with the clinical data when the *R*_sp_ of the chest was 10.4–15.4 Mcps, the *R*_sp_ of the abdomen was 11.0–16.6 Mcps, and the *R*_sp_ of the chest to abdomen was 21.4–32.1 Mcps. On the other hand, *R*_t+s_ showed some discrepancies between each phantom type and the clinical data. However, Thorax phantom was more similar to the clinical data than the two NEMA body phantoms. When phantoms simulating the human brain were placed outside the FOV, NEMA_Brain and Thorax_Brain data matched the clinical data well. The mean *R*_sp_ values of the chest, abdomen, and chest to abdomen in the clinical data were 12.9 Mcps, 13.7 Mcps, and 26.6 Mcps, respectively. Furthermore, Table 3 shows the percentage change in *R*_t+s_ for each phantom and *R*_t+s_ for clinical data at mean *R*_sp_. Chest to abdomen had the largest percentage change in NEMA and the smallest in Thorax_Brain. When the *R*_sp_ of the NEMA body phantom was 26.6 Mcps, the mean *R*_sp_ of the clinical data, the background radioactivity of the NEMA body phantom was found to be 4.52 kBq/mL. On the other hand, the background radioactivity of Thorax phantom was 2.48 kBq/mL. Background radioactivity estimated from count rates was more similar to patient data for Thorax phantom than for the two NEMA body phantoms.

**Table 3:** Percentage change in *R*_t+s_ for each phantom and *R*_t+s_ for clinical data at mean *R*_sp_

Phantom^a^	Percentage (%)		
	Chest	Abdomen	Chest to abdomen
NEMA	31.4	16.3	26.4
NEMA_Brain	− 5.7	− 0.3	− 1.2
Thorax	14.2	12.7	16.5
Thorax_Brain	− 9.7	4.7	0.5

^a^The phantoms are: NEMA denoting the two NEMA body phantoms; NEMA_Brain denoting the NEMA_Cylinder phantom; and Thorax_Brain denoting the Thorax_Cylinder phantom

**Fig. 5:**
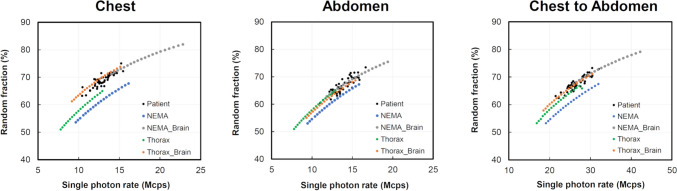
The correlation between the single photon rates of each phantom and the random fraction

The comparison with clinical data of RFs correlated with *R*_sp_ obtained from the four types of phantoms is made in Fig. 5. The RF was lowest for NEMA, and it was difficult to reproduce the RF obtained from the human body using only NEMA and Thorax. The RF increased when CCF Cylinder phantom, which simulates the human brain, was placed in the imaging FOV. This showed that NEMA_Brain and Thorax_Brain were in good agreement with the RF of the patients. These results showed a similar trend to the results presented in Fig. 4.

**Fig. 6:**
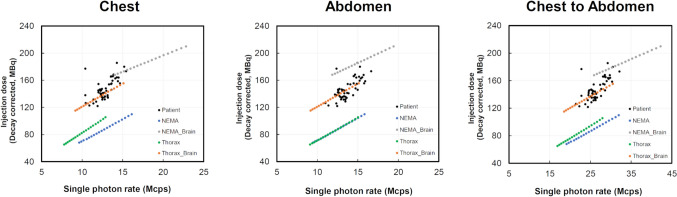
The correlation between the single photon rates of each phantom type and the injection dose (decay corrected, MBq)

The correlation between *R*_sp_ obtained from phantom and clinical data and actual dose corrected for decay at the PET scan start time from FDG administration is shown in Fig. 6. From patient data, for the chest to abdomen, based on the mean *R*_sp_ of 26.6 Mcps, the actual injection dose was 147.8 MBq. For NEMA_Brain and Thorax_Brain, when the mean chest to abdomen *R*_t+s_ matched the patient data, the actual injection dose for NEMA_Brain was 123.3 MBq and that for Thorax_Brain was 137.1 MBq.

### Image quality

**Fig. 7:**
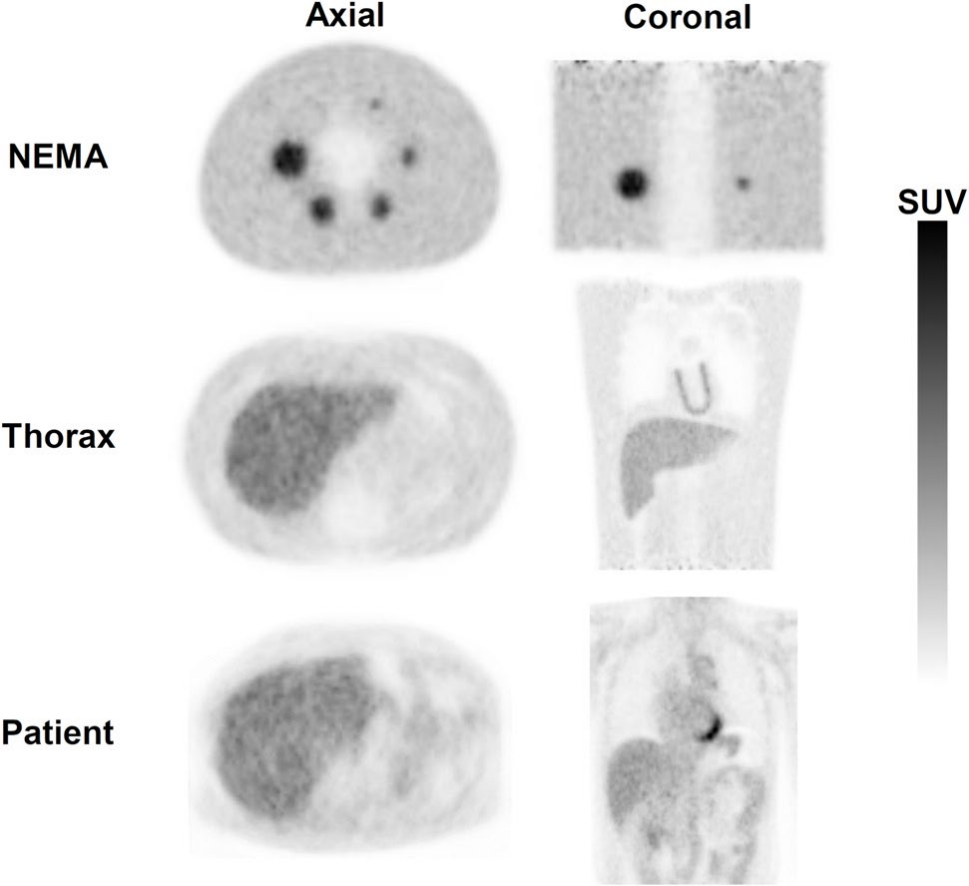
Comparison of PET images of patients and the NEMA body phantom and Thorax phantom obtained under the same image reconstruction conditions

Figure 7 shows PET images reconstructed under the same image reconstruction conditions showing the same mean *R*_sp_ of the chest to abdomen for the patient data as for Thorax phantom and the two NEMA body phantoms. Compared to the NEMA body phantoms, Thorax phantom visually reproduced the radioactivity distribution that more closely simulated the human body.

The results of NEC_patient_, NEC_density_, and *liver SNR*, physical indices of PET image quality obtained from the Thorax phantom data and clinical data, are shown in Table 4. The NEC_patient_ obtained from Thorax phantom was 1.4% higher than the mean obtained from the patient data. NEC_density_ was 15.0% lower than the mean value obtained from patient data. The mean *liver SNR* obtained by the OSEM method was 14.8% higher for Thorax phantom and that obtained by Q.Clear was 10.0% lower than the patient data.

**Table 4:** PET image quality parameters obtained for Thorax and patients (*n* = 51)

Parameters	Thorax	Patient (*n* = 51) mean ± SD
NEC_patient_	21.3	21.0 ± 2.5
NEC_density_	0.364	0.428 ± 0.1
Liver SNR (OSEM)	12.4	10.8 ± 1.5
Liver SNR (Q.Clear)	15.3	17.0 ± 3.8

## Discussion

In this study, the technical usefulness of a novel anthropomorphic phantom capable of reproducing the thorax phantom region of the human body was evaluated. Count rate characteristics showed a tendency for the commercial Thorax phantom to match the count rate obtained from human bodies compared to the NEMA body phantom. Furthermore, it was found that the human body count rate was more consistent with the human body count rate when a radiation source simulating the human brain was placed in the imaging FOV. Previous studies have been able to match count rates obtained from anthropomorphic phantoms and human subjects, but this has not been adequately validated in the thoracoabdominal imaging area. In addition, it has been difficult to evaluate image quality for the previously reported anthropomorphic phantoms under the same conditions as in a clinical study. In this work, image quality indices such as NEC_patinet_, NEC_density_ and liver SNR were calculated and evaluated under the same conditions as an actual clinical study, using the Thorax phantom that almost ideally reproduces the structure and count rate of the human body.

The *R*_sp_-based method reported by Smith et al. [19] is useful for validation of count rate characteristics derived from human and phantom data. To investigate the radioactivity concentration in the human body, ROIs are often placed at specific locations and evaluated, but it is difficult to accurately evaluate the background and other radioactivity concentrations, because the radioactivity concentration in the human body has a non-uniform distribution. It is well known that *R*_sp_-based methods to reproduce the radioactivity concentration in the human body are effective. Here, in addition to *R*_p_ and *R*_r_ count rates based on *R*_sp_ in the chest and abdomen, obtained with reference to the method reported by Lartizien et al. [20], *R*_t+s_ based on *R*_sp_ was also evaluated with reference to the method reported by Inoue et al. [14]. Not only the chest or abdomen (bed position-1), but also the imaging range of the chest and abdomen (bed position-2) were evaluated. As shown in Fig. 4, the *R*_p_ and *R*_r_ obtained for the patients and phantoms were in good agreement. This was in agreement with the previous results of [20]. *R*_t+s_ count rates based on *R*_sp_ in clinical data were more consistent with human body count rates for Thorax phantom than for NEMA body phantoms. This may be due to the fact that Thorax phantom was designed to simulate the size of each organ of the human body, as shown in Fig. 1, and the radioactivity can be included in the lungs, liver, kidneys, and background with a high degree of freedom. On the other hand, in the case of the NEMA body phantoms, only the hot sphere and background region can contain radiopharmaceuticals due to structural limitations, which may have made it difficult to reproduce the non-uniform radioactivity concentration distribution in the human body.

In general, the brain is known to be the highest physiological accumulation region for FDG. In this study, the CCF Cylinder phantom used in the performance evaluation of PET scanners was placed in the imaging FOV of a radiation source simulating the human brain. The results showed that NEMA_Brain and Thorax_Brain had increased RF, and they matched the count rates in patients better than NEMA and Thorax did, as shown in Fig. 5. The reason for this was that in the clinical study, FDG shows a high accumulation in the brain, which is influenced by the number of random coincidences in the imaging FOV. This was consistent with the report by Inoue et al. [14] who found that an anthropomorphic phantom could be made to agree with human body count rates by taking into account the effects of attenuation and scatter inside and outside the FOV. In the present experiment, the CCF Cylinder phantom was placed 15 cm away from the NEMA body phantoms and Thorax phantom in the head-side direction, respectively, which tended to increase the number of random coincidences, an image quality degradation factor, in agreement with the clinical data. Therefore, in clinical studies, the thorax phantom of the human body is expected to have a higher random coincidence count than the abdomen due to the effect of out-of-field scattered radiation from the brain, which has a relatively high physiological accumulation of FDG. The *R*_t+s_ were also higher in the abdomen than in the chest. This was consistent with reports of higher NEC peaks in the abdomen than in the chest, and similar to the validated results of Lartizien et al. [20].

The correlation between the *R*_sp_-based patient dose and the actual injection dose of each phantom type was studied, as shown in Fig. 6, and Thorax_Brain gave the best agreement with the clinical data. On the other hand, NEMA_Brain had good agreement for the count rate characteristics in Figs. 4 and 5, but diverged for the actual injection dose. The reason for this may be that the distribution of radioactivity concentrations in the human body is non-uniform, so a simple structure such as the NEMA body phantom cannot reproduce the distribution of radioactivity concentrations in the body. Thorax phantom can contain the same radioactivity as the human body for each organ, which is thought to be a factor in its ability to reproduce the distribution of radioactivity concentration in the body more closely.

The NEC_patient_, NEC_density_, and liver SNR physics indices were calculated to assess the image quality of PET images obtained from phantom and clinical data. NEC_patient_ and NEC_density_ have the advantage that they are calculated from count data before image reconstruction and are therefore not influenced by image reconstruction conditions. On the other hand, these image quality indicators have the disadvantage that they do not take into account factors of image reconstruction. Liver SNR is calculated by placing ROIs on PET images obtained by image reconstruction, and therefore, it includes an effect from image reconstruction conditions. These PET image quality indices are defined in Japanese guidelines, where NEC_patient_ > 13 (Mcounts/m), NEC_density_ > 0.2 (kcounts/cm^3^) and liver SNR > 10 are acceptable reference values [17]. The mean values of NEC_patient_, NEC_density_, and liver SNR obtained in the clinical data were above the reference values, but all values obtained in the Thorax phantom was close to the reference values. The commercial Thorax phantom is a new image quality assessment tool that reproduces real clinical situations, as it not only reproduces counting rates, but also allows PET image quality to be assessed under the same conditions.

As shown in Table 4, the obtained NEC_patient_ values were in good agreement between patients and phantom types. The reason for this is that the NEC_patient_ is calculated by normalizing NEC by the imaging length, so if the clinical and phantom data count rates match, the NEC_patient_ is also expected to match. On the other hand, NEC_density_ differed by 15.0% between clinical and phantom data. The reason for this is that NEC_density_ is calculated by normalizing the NEC by volume, so even if the count rates are matched, if the volumes of the human body and the phantom are different, NEC_density_ will not match. In fact, the volumes obtained in the assessed range differed by 15.5% between Thorax phantom and patients. Liver SNR values showed that Q.Clear resulted in a higher liver SNR compared to OSEM. This is consistent with the results reported by Teoh et al. [21] of Q.Clear giving a higher SNR than OSEM.

The present study had some limitations. It was validated using only one PET scanner, and it is not known whether similar results can be achieved using a different scanner, as the count rate obtained depends on the scanner performance. In addition, the PET image quality indices here only examined NEC_patinet_, NEC_density_, and liver SNR and they did not assess the detectability of tumor lesions or quantitative terms such as SUV.

## Conclusion

The Thorax results were in better agreement with count rates obtained from clinical data than a conventional NEMA body phantom and the former was able to better simulate the distribution of radioactivity concentrations in the human body by reproducing that distribution outside the FOV. Furthermore, Thorax phantom enabled PET image quality assessment using the same image quality metrics as for the human body, which is difficult to achieve with the conventional NEMA body phantom, suggesting that this anthropomorphic phantom is an effective PET image quality assessment tool that reproduces clinical conditions.

## Data Availability

The datasets used and/or analyzed during the current study are available from the corresponding author upon reasonable request.

## References

[1] Rohren EM, Turkington TG, Coleman RE. Clinical applications of PET in oncology. Radiology. 2004;231(2):305–32. 15044750 10.1148/radiol.2312021185

[2] Weber WA. Assessing tumor response to therapy. J Nucl Med. 2009;50(Suppl 1):1S-10S. 19380403 10.2967/jnumed.108.057174

[3] Lodge MA, Wahl RL. Practical PERCIST: a simplified guide to PET response criteria in solid tumors 1.0. Radiology. 2016;280(2):576–84. 26909647 10.1148/radiol.2016142043 PMC4976461

[4] Meikle SR, Sossi V, Roncali E, Cherry SR, Banati R, Mankoff D, et al. Quantitative PET in the 2020s: a roadmap. Phys Med Biol. 2021;66(6):06RM1. 10.1088/1361-6560/abd4f7 PMC9358699 33339012

[5] O’connor JP, Aboagye EO, Adams JE, Aerts HJ, Barrington SF, Beer AJ, et al. Imaging biomarker roadmap for cancer studies. Nat Rev Clin Oncol. 2017;14(3):169–86. 27725679 10.1038/nrclinonc.2016.162 PMC5378302

[6] Boellaard R. Standards for PET image acquisition and quantitative data analysis. J Nucl Med. 2009;50(Suppl 1):11S-20S. 19380405 10.2967/jnumed.108.057182

[7] Fahey FH, Kinahan PE, Doot RK, Kocak M, Thurston H, Poussaint TY. Variability in PET quantitation within a multicenter consortium. Med Phys. 2010;37:3660–6. 20831073 10.1118/1.3455705 PMC2905446

[8] Kinahan PE, Perlman ES, Sunderland JJ, Subramaniam R, Wollenweber SD, Turkington TG, et al. The QIBA profile for FDG PET/CT as an imaging biomarker measuring response to cancer therapy. Radiology. 2020;294(3):647–57. 31909700 10.1148/radiol.2019191882 PMC7053216

[9] Graham MM, Wahl RL, Hoffman JM, Yap JT, Sunderland JJ, Boellaard R, et al. Summary of the UPICT protocol for ^18^F-FDG PET/CT imaging in oncology clinical trials. J Nucl Med. 2015;56(6):955–61. 25883122 10.2967/jnumed.115.158402 PMC4587663

[10] Senda M. Standardization of PET imaging and site qualification program by JSNM: collaboration with EANM/EARL. Ann Nucl Med. 2020;34(11):873–4. 32939687 10.1007/s12149-020-01518-y

[11] Brambilla M, Matheoud R, Secco C, Sacchetti G, Comi S, Rudoni M, et al. Impact of target-to-background ratio, target size, emission scan duration, and activity on physical figures of merit for a 3D LSO-based whole body PET/CT scanner. Med Phys. 2007;34(10):3854–65. 17985631 10.1118/1.2776242

[12] Doshi NK, Basic M, Cherry SR. Evaluation of the detectability of breast cancer lesions using a modified anthropomorphic phantom. J Nucl Med. 1998;39(11):1951–7. 9829588

[13] Matheoud R, Secco C, Della Monica P, Leva L, Sacchetti G, Inglese E, et al. The effect of activity outside the field of view on image quality for a 3D LSO-based whole body PET/CT scanner. Phys Med Biol. 2009;54(19):5861–72. 19759405 10.1088/0031-9155/54/19/013

[14] Inoue K, Sato T, Kitamura H, Hirayama A, Kurosawa H, Tanaka T, et al. An anthropomorphic pelvis phantom for optimization of the diagnosis of lymph node metastases in the pelvis. Ann Nucl Med. 2009;23(3):245–55. 19319630 10.1007/s12149-009-0229-5

[15] Sunderland JJ, Christian PE. Quantitative PET/CT scanner performance characterization based upon the society of nuclear medicine and molecular imaging clinical trials network oncology clinical simulator phantom. J Nucl Med. 2015;56(1):145–52. 25525180 10.2967/jnumed.114.148056

[16] Jha AK, Mithun S, Puranik AD, Purandare NC, Shah S, Agrawal A, et al. Performance characteristic evaluation of a bismuth germanate-based high-sensitivity 5-ring discovery image quality positron emission tomography/computed tomography system as per National Electrical Manufacturers Association NU 2–2012. World J Nucl Med. 2019;18(4):351–60. 31933550 10.4103/wjnm.WJNM_72_18 PMC6945355

[17] Fukukita H, Suzuki K, Matsumoto K, Terauchi T, Daisaki H, Ikari Y, et al. Japanese guideline for the oncology FDG-PET/CT data acquisition protocol: synopsis of version 2.0. Ann Nucl Med. 2014;28:693–705. 24859759 10.1007/s12149-014-0849-2 PMC4332454

[18] Matsumoto K, Endo K. Development of analysis software package for the two kinds of Japanese Fluoro-D-glucose-positron emission tomography guideline. Jpn J Radiol Technol. 2013;69:648–54. 10.6009/jjrt.2013_jsrt_69.6.648 23782777

[19] Smith RJ, Adam LE, Karp JS. Methods to optimize whole body surveys with the C-PET camera. In: Proceedings of the IEEE nuclear science symposium and medical imaging conference (Seattle, WA, 1999), vol. 3. Los Alamitos: IEEE; 1999, pp.1197–1201.

[20] Lartizien C, Comtat C, Kinahan PE, Ferreira N, Bendriem B, Trébossen R. Optimization of injected dose based on noise equivalent count rates for 2- and 3-dimensional whole-body PET. J Nucl Med. 2002;43(9):1268–78. 12215569

[21] Teoh EJ, McGowan DR, Macpherson RE, Bradley KM, Gleeson FV. Phantom and clinical evaluation of the Bayesian penalized likelihood reconstruction algorithm Q. Clear on an LYSO PET/CT system. J Nucl Med. 2015;56(9):1447–52. 26159585 10.2967/jnumed.115.159301 PMC4558942

